# Low Muscle Strength Assessed with Dynamometry in Elderly Polypathological Patients with Acute Heart Failure: PROFUND-IC Registry

**DOI:** 10.3390/jcm13164873

**Published:** 2024-08-18

**Authors:** Alicia Guzmán-Carreras, Jorge San Miguel-Agudo, Mateo Paz-Cabezas, Máximo Bernabeu-Wittel, Nuria Muñoz-Rivas, Beatriz Sánchez-Sauce, Fernando Aguilar-Rodríguez, Luis Cabeza-Osorio, Emmanuel Andrès, Noel Lorenzo-Villalba, Manuel Méndez-Bailón

**Affiliations:** 1 Servicio de Medicina Interna, Hospital Clínico San Carlos, 28040 Madrid, Spain; 2 Faculty of Medicine. Universidad Complutense de Madrid, Hospital Clínico San Carlos, 28040 Madrid, Spain; 3 Unidad de Apoyo Metodológico a la Investigación, Servicio de Medicina Preventiva, Hospital Clínico San Carlos, 28040 Madrid, Spain; 4 Servicio de Medicina Interna, Hospital Virgen del Rocío, 41013 Sevilla, Spain; 5 Servicio de Medicina Interna, Hospital Infanta Leonor, 28031 Madrid, Spain; 6 Servicio de Medicina Interna, Hospital Fundación Alcorcón, 28922 Madrid, Spain; 7 Servicio de Medicina Interna, Hospital 12 de Octubre, 28041 Madrid, Spain; 8 Servicio de Medicina Interna, Hospital del Henares, 28822 Madrid, Spain; 9 Servicio de Medicina Interna, Hopitaux Universitaire de Strasbourg, 67000 Strasbourg, France

**Keywords:** heart failure, muscle strength, dynamometry

## Abstract

**Background:** Sarcopenia is a comorbidity associated with heart failure, which aggravates its prognosis. **Objectives:** To analyze the differential characteristics of polypathological patients with acute heart failure (AHF) based on the presence of low muscle strength, as well as to study whether this condition is associated with a worse prognosis. **Methods:** An observational study of 377 patients with a diagnosis of acute heart failure from the prospective multicentric PROFUND-IC registry was carried out. The main variable is low muscle strength, which is assessed with dynamometry or prehensile strength. Epidemiological and anthropometric characteristics, as well as associated comorbidities, were analyzed. Likewise, the etiology of the AHF episode, the number of admissions in the previous year, and the NYHA scale were also included. Finally, scores on functionality, treatment established, and mortality and readmission rates were studied. Quantitative variables are described as mean, and standard deviation, and qualitative variables are expressed as absolute numbers and percentages. A descriptive and bivariate analysis was performed according to the presence of low muscle strength (handgrip <27 kg in men and <16 kg in women), using the Welch test for quantitative measures and Chi-square for qualitative variables. In addition, Kaplan-Meier curves of readmission and mortality and a logistic regression analysis were also performed. **Results:** 377 patients were included (56% female, mean age 83 years). 310 (82.23%) had low muscle strength. Those with low muscle strength were older (84 vs. 78 years, *p* < 0.001), with more cognitive impairment (11.9% vs. 0%, *p* = 0.021), worse functional class (*p* = 0.016), lower scores in the Barthel index and Rockwood scale (*p* < 0.001), and higher in the PROFUND index (*p* < 0.001). They had higher rates of readmission and mortality without statistically significant differences. The PROFUND index is significantly associated with low muscle strength (OR 1.19, CI (1.09–1.31), *p* < 0.001). **Conclusions:** Elderly polypathological patients with acute heart failure and low muscle strength have a higher PROFUND index and a lower probability of survival per year.

## 1. Introduction

Heart failure (HF) is a clinical syndrome characterized by a structural or functional cardiac disorder that causes reduced cardiac output at rest or during exercise, usually with elevated intracardiac pressures [1]. It is classified according to left ventricular ejection fraction (LVEF) as HF with reduced LVEF (≤40%), HF with mildly reduced LVEF (41–49%), and HF with preserved LVEF (≥50%) [2].

HF is a very common and conditioning disease. In Spain, it affects 1.89% of the population and causes 2.78 new cases per 1000 subjects/year [3]. Moreover, 3% of hospital admissions are due to HF, and it is the first cause of hospitalization in those over 65 years of age [3].

Mortality due to HF in 2021 in Spain was 29.3% in acute heart failure and 6.4% in chronic heart failure [3]. Noteworthy, approximately one-third of patients will have died within one year after an admission for acute HF, and one of every two will have died or will have needed readmission [4].

Therefore, it is a prevalent disease that is also often accompanied by other comorbidities that make patient management difficult. Among them, sarcopenia stands out [5].

Sarcopenia is a skeletal muscle disease that results in a reduced amount of muscle mass and a loss of muscle function. Like HF, it is an age-dependent pathology. Its prevalence varies between 6 and 22% of the population over 65 years of age, affecting 14–38% of institutionalized patients and 10% of hospitalized ones [6].

The SARC-F questionnaire is used as a screening tool to evaluate the symptoms of muscle weakness [7]. In patients with suspected sarcopenia (SARC-F questionnaire ≥ 4), we should perform muscle strength tests: manual dynamometry (Handgrip) for the upper body and Sit-to-stand test for the lower body. The cut-off points of Handgrip are 27 kg for men and 16 kg for women [7]. Results below these cut-off points are patients with probable sarcopenia. The diagnosis of confirmed sarcopenia is made when pathological results are obtained in muscle mass estimation techniques (dual-energy X-ray absorptiometry (DXA)) [7]. Finally, severe sarcopenia is defined as confirmed sarcopenia accompanied by a decrease in physical performance [7].

The prevalence of sarcopenia among patients with heart failure reaches 34% [8]. There is an interplay between the pathophysiological pathways involved in HF and sarcopenia [8]. Several factors related to HF, like hormonal changes, malnutrition, inflammation and oxidative stress, low muscle blood flow, endothelial dysfunction, and physical inactivity, may contribute to the development of sarcopenia [9]. At the same time, sarcopenia significantly affects physical performance because of abnormalities in peripheral blood flow and skeletal muscle, which is one of the most important prognosis factors in HF. In summary, the adaptive changes secondary to these pathologies lead to worse functionality and prognosis of patients and condition the relationship between both entities [8,9,10].

In conclusion, sarcopenia is a comorbidity frequently associated with heart failure. It seems to cause a worse quality of life and prognosis. The aims of this study were (1) to analyze the differential characteristics of polypathological patients admitted for acute heart failure with low muscle strength measured by dynamometry versus those without it, (2) to study whether the loss of muscle strength obtained by dynamometry below the reference limit values (probable sarcopenia) is a prognostic factor related to increased mortality or readmission rates in patients with acute heart failure, (3) to evaluate the relationship between loss of strength measured by dynamometry (hand-grip) and NYHA functional classification.

## 2. Methods

### 2.1. Study Design

This is a retrospective observational study of 377 polypathological patients admitted to Internal Medicine with a primary diagnosis of acute heart failure. Data were collected from 1 October 2020, to 31 May 2023, from the PROFUND-IC multicenter registry. PROFUND-IC registry was originally created as a prospective cohort study with the collaboration of the Working Group on Heart Failure and Atrial Fibrillation and the Group of Polypathological Patients and Advanced Age of the Spanish Society of Internal Medicine.

### 2.2. Population Study

The registry includes 748 patients registered between October 2020 and 31 May 2023. The 377 who had dynamometry performed were selected.

#### The Inclusion Criteria Are

▪ Age over 18 years.
▪ Have a primary diagnosis of acute heart failure (HF) as the reason for admission, including episodes of de novo HF and decompensation of chronic heart failure.
▪ NTproBNP > 1500 pg/mL at the time of admission.
▪ Fulfill the criterion of a polypathological patient, defined as the presence of 2 or more concomitant chronic diseases.
▪ To have performed dynamometry (Handgrip) as a measure of muscle strength at the time of admission.
▪ To have accepted and signed the informed consent by the patient or a legal representative.
▪ Complete clinical follow-up for one year.

Exclusion criteria were refusal to sign the informed consent form or patients without dynamometry performed.

### 2.3. Variables Studied

The main determinant variable of low muscle strength is dynamometry or prehensile strength, using a cut-off point lower than 27 kg for men and 16 kg for women as pathologic [7]. Secondary variables included epidemiological characteristics such as sex and age, as well as anthropometric values (weight, height, and body mass index (BMI)). As the patients were polypathological, previous comorbidities such as arterial hypertension, diabetes mellitus, dyslipidemia, cognitive impairment, atrial fibrillation, chronic renal disease, chronic respiratory disease, history of cerebrovascular disease, presence of active solid or hematological neoplasm, and chronic osteoarticular disease were considered.

For heart failure, variables such as the etiology of the episode of decompensation, score on the Everest scale, the number of admissions in the last year, functional class according to the NYHA (New York Heart Association) scale, and left ventricular ejection fraction (LVEF) were analyzed. Functional variables (Short Physical Performance Battery (SPPB) and Barthel scores), nutritional screening (Mini Nutritional Assessment Short Form (MNA-SF)), and life expectancy in multi-pathological patients (PROFUND Index and Rockwood scale) were also recorded.

Analytical variables were also studied (hemoglobin (g/dL), lymphocyte count (×10^6^/L), serum creatinine (mg/dL), LDL cholesterol (mg/dL), albumin (g/dL), NT-proBNP (pg/mL) and Ca 125 (U/mL). The treatment administered was also included, such as the maximum daily dose of furosemide during admission (mg/day), protein supplements, corticosteroids, or morphine. Finally, prognostic variables were investigated, such as the percentage of deaths both during baseline admission and in the course of follow-up, as well as the 12-month readmission rate.

### 2.4. Statistical Study

Quantitative variables are collected as mean and standard deviation (or as median and interquartile range if the variable did not conform to normality). Likewise, qualitative variables are described as absolute numbers and percentages.

A descriptive analysis of the sample was performed, as well as a bivariate analysis, according to the presence of low muscle strength defined as less than 27 kg for men and 16 kg for women. For the comparison of quantitative variables, the Welch test was used, and the Chi-square test was used for qualitative ones. The Wilcoxon test and the Fisher test were applied, respectively, in those cases in which the variables did not adjust to normality.

The estimation of the probability of survival and readmission during follow-up (12 months) was carried out using the Kaplan-Meier method. In the case of comparisons between groups, the statistical test used was the log-rank test.

Furthermore, multivariable logistic regression was performed following the stepwise backward methodology to obtain a final prediction model of low muscle strength measured by dynamometry. The model is described by a forest plot, including each variable with its odds ratio and the ROC curve with the area under the curve.

Finally, to determine the optimal cutoff point for the PROFUND index that would predict low muscle strength in patients with the best balance of sensitivity and specificity, we used the “cutpointr” package for R. A ROC curve was created to predict the muscle strength according to the PROFUND index, and an optimal cutoff point was obtained according to the Youden score.

The required alpha error was less than 5% with a power of 0.8. Statistical analysis was performed using the R statistical program and Rstudio v 4.3.

### 2.5. Work Methodology

Regarding the methodology of the study, the first step was for the patient to accept and sign the informed consent. Subsequently, a clinical assessment was carried out, including the anthropometric parameters and functionality scales previously mentioned. A blood test was also required (hemoglobin, lymphocyte count, serum creatinine, LDL cholesterol, albumin, NT-proBNP, and Ca 125. To perform the dynamometry, the patient had to be seated, with the dominant arm flexed at 90 degrees and holding the dynamometer. Subsequently, they must perform a maximum grip for three to five seconds with a recovery time of 30 s in three attempts, taking into account the best of them [7]. A handheld digital dynamometer available at the hospital was used for this purpose. The process had to be carried out in the first 72 h of admission. All those responsible for incorporating patients into the study had been trained in performing the technique. This process was performed in the Internal Medicine hospitalization ward, requiring additional materials such as a dynamometer, a computer with access to the hospital’s computer program, and an analysis laboratory. Once the data were collected, they were incorporated into the anonymized database and subsequently analyzed statistically according to the tests explained before.

### 2.6. Ethical Aspects

The PROFUND-IC registry was approved by the Clinical Research Ethics Committee of the Hospital Fundación Alcorcón (Annex I). The work complies with the ethical requirements of the Declaration of Helsinki. The patients, or failing this, their legal representative, in addition to being informed verbally in detail about the registry, signed their written Informed Consent before being included in the study. There is no conflict of interest on the part of the authors.

## 3. Results

A total of 377 patients were included, of whom 310 (82.23%) presented low muscle strength measured by dynamometry. Of these, 211 were women (55.97%), and the mean age was 83 years. The mean weight was 73 kg (±17), and the height was 160 cm (±9). Most were overweight according to BMI (median 27 kg/m^2^ (24–32)). The most frequently identified comorbidities were arterial hypertension (343, 90.98%) and atrial fibrillation (286, 75.86%) (Table 1).

Regarding the etiology of HF, the most frequent cause implicated as responsible for the episode of decompensation was hypertension (154, 40.85%). Functional class, according to the NYHA scale, was type II in 169 patients (44.83%) and type III in 179 (47.48%). The mean LVEF was 52 (±13) %. On arrival, patients had a median NTproBNP of 5500 pg/mL (2956–10,493) and a CA-125 of 41 U/mL (20–89) (Table 1).

Regarding functional variables, the median Barthel index was 75 (50–95), and the SPPB was 3 (0–6). According to the Rockwood scale, most patients (119, 31.56%) required help in both instrumental (IADL) and basic activities of daily living (BADL). The PROFUND index was used as a frailty variable, with a median of 6 (3–10) (Table 1).

**Figure 1:**
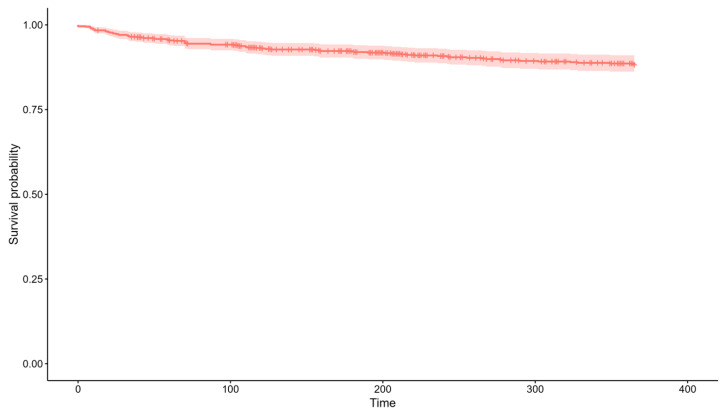
Descriptive of mortality at twelve months in the sample (N = 377). Twenty-two patients (5.84%) died during admission and 36 (9.55%) more during the following 12 months.

**Figure 2:**
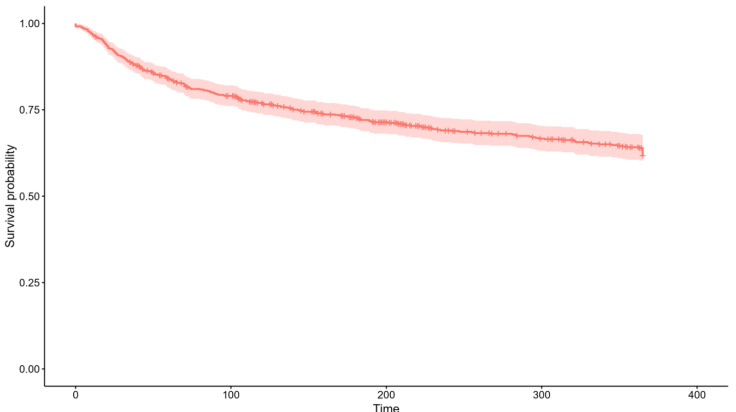
Descriptive of 12-month readmissions in the sample (N = 377). One hundred thirty-two patients (35.01%) were readmitted during follow-up.

During admission, the median maximum dose of administered furosemide was 80 mg/day (60–120). 99 (26.26%) patients received corticosteroids, and 37 (9.81%) took protein supplements. Finally, prognostic variables were collected. Twenty-two patients (5.84%) died during admission and 36 (9.55%) more during the following 12 months (Figure 1). In addition, 132 (35.01%) were readmitted during follow-up (Figure 2).

In the comparative analysis between those with low muscle strength versus those with normal muscle strength, it is observed that the subgroup of patients with normal muscle strength was younger (78 versus 84 years, *p* < 0.001). There are no statistically significant differences in terms of sex (Table 2).

Patients with low muscle strength had lower weight (72 vs. 78 kg, *p* = 0.016), shorter height (160 vs. 164 cm, *p* = 0.003), and lower BMI (27 vs. 29 kg/m^2^) compared to those with normal muscle strength. The difference in BMI was not significant (Table 2).

Concerning the comorbidities studied, statistically significant differences were found only in cognitive impairment between the two groups. No patient with normal muscle strength suffered cognitive impairment compared to 37 patients (11.94%) with decreased muscle strength who did (*p* = 0.021) (Table 2).

Regarding the etiology of HF, no statistically significant differences were observed between the two groups. Nevertheless, although hypertension continued to be the most frequent cause of decompensation in both groups, those with normal muscle strength showed an increase in other etiologies, such as ischemia (29.85% vs. 17.74%). A statistically significant higher rate of admissions in the previous year was also observed in the group with low muscle strength (35.16% of patients with low muscle strength were readmitted two or three times, compared to 28.36% with normal muscle strength) (*p* = 0.047) (Table 2).

Patients with low muscle strength also suffered worse functional class according to the NYHA scale (51.29% of patients with low muscle strength had NYHA III vs. 29.85% with normal muscle strength) (*p* = 0.016). Mean LVEF was lower in the group with normal muscle strength (48% vs. 52%, *p* = 0.033). Barthel index was lower in those with low muscle strength (70 vs. 95, *p* < 0.001), as well as the score on the SPPB test (2 vs. 5, *p* < 0.001) (Table 2).

With respect to the PROFUND index, higher scores are observed in those with low muscle strength (7 vs. 3, *p* < 0.001), so the probability of survival of them is lower. Again, both groups present statistically significant differences in the Rockwood scale, where patients with good condition and controlled disease were higher in the normal muscle strength group compared to those with low muscle strength (32.84% vs. 17.10%, (*p* < 0.001)), (Table 2).

The maximum daily dose of furosemide administered was higher in patients with normal muscle strength (120 mg/day) compared to patients with low muscle strength (80 mg/day) (*p* = 0.047). In addition, the group with low muscle strength received more corticosteroids (29.03% vs. 13.43%, *p* = 0.003) and protein supplements (10.65% vs. 5.97%, *p* = 0.5) (Table 2).

**Figure 3:**
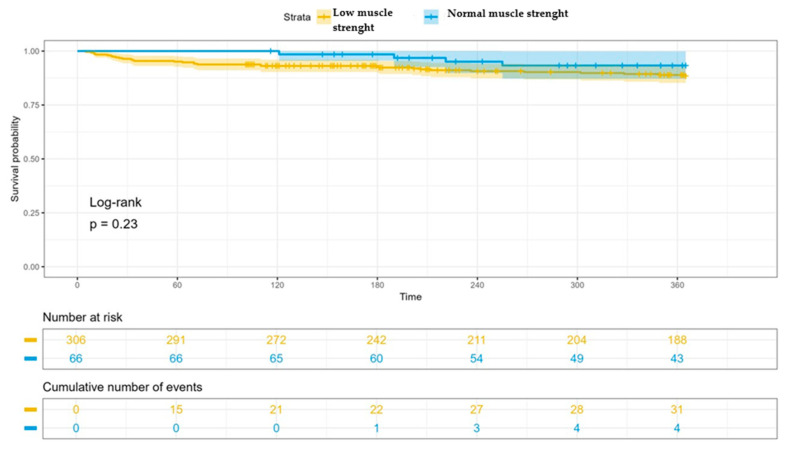
Mortality analysis during the 12-month follow-up in the group with normal muscle strength versus low muscle strength by dynamometry. Patients with normal muscle strength had lower mortality rates both during baseline admission (4.48% vs. 6.13%, *p* = 0.8) and follow-up (5.97% vs. 10.32%, *p* = 0.23).

**Figure 4:**
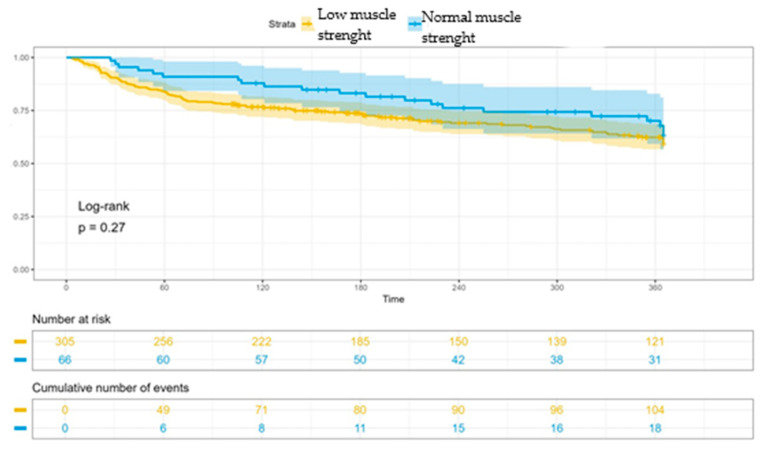
Analysis of readmission during 12-month follow-up in the group with normal muscle strength versus low muscle strength by dynamometry. Patients with low muscle strength have a higher readmission rate at 12 months (35.81% vs. 31.34%) (*p* = 0.27).

Finally, patients with normal muscle strength had lower mortality rates both during baseline admission (4.48% vs. 6.13%, *p* = 0.8) and follow-up (5.97% vs. 10.32%, *p* = 0.23), although this did not reach statistical significance (Figure 3). Consistent with the previous, patients with low muscle strength also have a higher readmission rate at 12 months (35.81% vs. 31.34%) (*p* = 0.27) (Figure 4).

**Figure 5:**
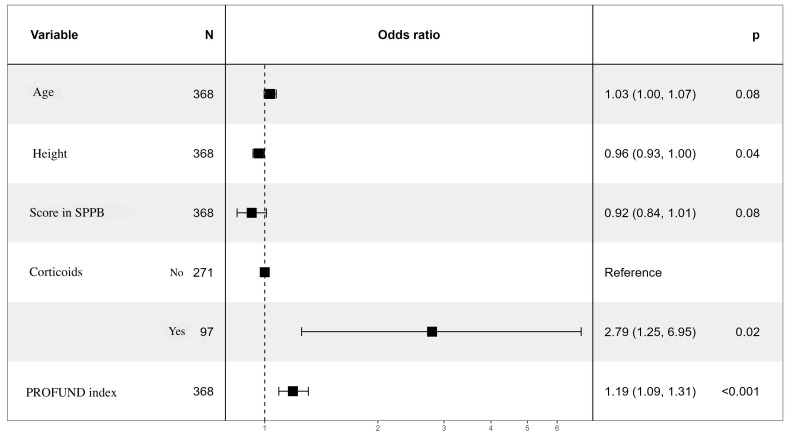
Forest Plot. Multivariate analysis. Variables associated with low muscle strength by dynamometry.

**Figure 6:**
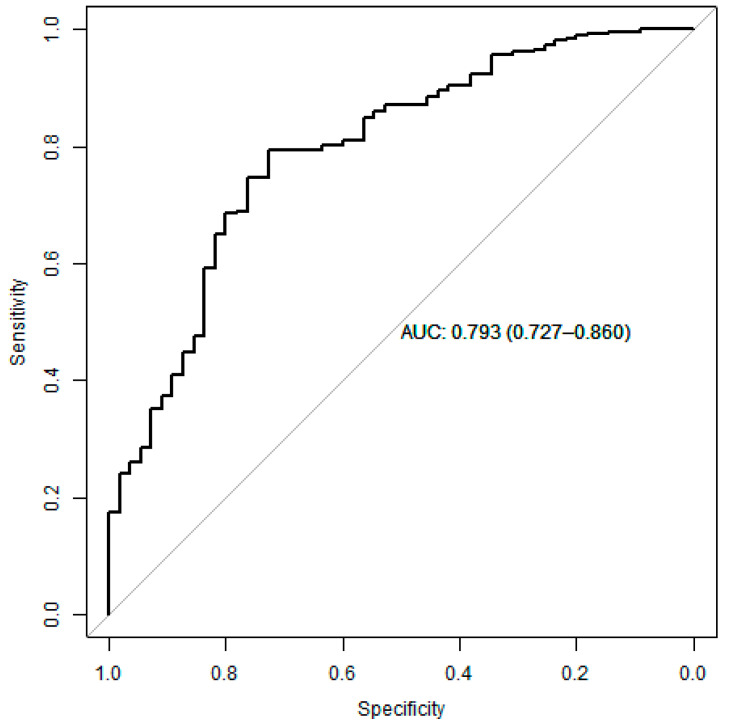
ROC curve of the low muscle strength prediction model created by multivariate analysis.

A multivariate analysis was performed to create a final predictive model of low muscle strength measured by dynamometry. The variables associated with low muscle strength were age, height, score in the SPPB test, corticosteroid use, and PROFUND index (Figure 5). Only corticosteroid administration (OR 2.79, CI (1.25–6.95), *p* = 0.02) and PROFUND index (OR 1.19, CI (1.09–1.31), *p* < 0.001) were independently and significantly associated with low muscle strength. The area under the curve of the final model is 0.793, with a confidence interval of 0.727–0.860 (Figure 6).

**Figure 7:**
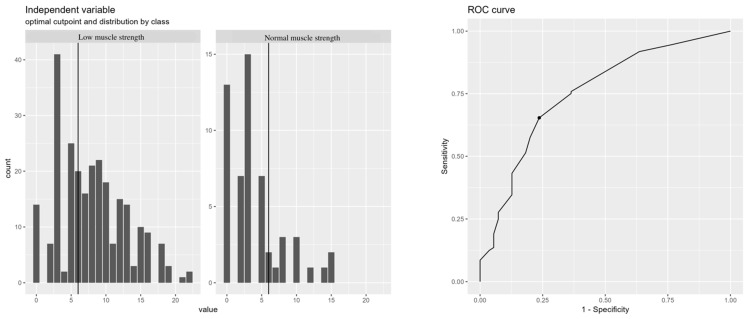
The cutoff point of the PROFUND index predicts low muscle strength. ROC curve predicting muscle strength according to PROFUND index.

The calculation of the optimal cutoff point set the value for PROFUND index on 6 or higher to discriminate between low or normal muscle strength, with a sensitivity of 0.65 and a specificity of 0.76 (AUC 0.751) (Figure 7).

## 4. Discussion

In this study, elderly polypathological patients with acute heart failure and low muscle strength measured by dynamometry had a higher Profund index and, therefore, a lower probability of survival at one year.

The results show that 82.23% of patients included had reduced muscle strength measured by dynamometry. The prevalence of sarcopenia in other studies is significantly lower (20% [11,12,13]–32.5% [14]). These differences could be explained by the pluripathology of the patients included, resulting in a more fragile and comorbid cohort. It could also be due to the lack of consensus on the definition of low muscle strength. In the present study, it is defined as dynamometry below 27 kg in men and 16 kg in women. The criteria used in the literature differ, finding bibliographies that follow this same cut-off point [13] but also others with different definitions [15]. Finally, in this study, we have considered low muscle strength measured by dynamometry, but to conclusively reach a diagnosis of sarcopenia, muscle mass should also be assessed. If both criteria were combined, the percentage of sarcopenic patients could be lower. In fact, in one of the articles consulted, 61.7% of the patients presented a pathological hand-grip test, but only 19.9% of them met sarcopenia criteria when muscle mass was quantified [13].

The sample is composed of 55.97% women and 44.03% men. In the literature, women represent a lower percentage, around 30% [11,12,14], reaching 41.6% in some studies [13]. In others, the sample studied is only made up of men [15]. The population included here is predominantly polypathological and elderly, similar to the profile of the typical patient encountered in the hospitalization wards of Internal Medicine. Therefore, given their longer life expectancy, the female sex predominates. In addition, the majority have preserved left ventricular ejection fraction, which is also more common in women.

Furthermore, in the comparative analysis between low muscle strength and normal muscle strength, it is observed that the subgroup of patients with normal muscle strength is younger (78 versus 84 years of age). This agrees with previous evidence since the prevalence of sarcopenia is higher in those who are older [13,14]. Nevertheless, the age of the patients included differs according to the study, and in some of it, the age of those affected by this pathology is similar to ours (82 years) [13], while in others, the population is much younger, 66 years [11]. One of the strengths of the study is to include patients from real clinical practice, hence the older age compared to the literature.

Regarding anthropometric variables, the presence of a lower BMI in subjects with low muscle strength is similar to the described bibliography [13,14]. This trend is observed but we have not reach statistical significance probably because of the low sample size.

Analyzing the comorbidities associated with heart failure, only cognitive impairment is statistically significantly greater in those with low muscle strength. Cognitive impairment affects patients with heart failure in non-negligible numbers, which range between 30% and 80% [16], greater than that evidenced. This could be due to the frequent underdiagnosis of this pathology. In addition, sarcopenia is associated with an increased risk of cognitive impairment [17]. Therefore, it seems reasonable that a higher percentage of cognitive impairment is observed in patients with low muscle strength.

Concerning other comorbidities, no statistically significant differences have been found according to muscle strength. Some studies report a higher rate of active malignant disease in groups with low muscle strength [13,14], as well as a higher prevalence of smokers and ex-smokers [13] and the use of implantable devices in this group [13]. In the literature consulted, other more frequent comorbidities, such as diabetes, dyslipidemia, or hypertension, persist without significant differences according to muscle strength [13,14]. However, there is again a link described between sarcopenia and different pathologies, such as type II diabetes mellitus [18]. The failure to find significant differences is probably due to the small sample size in addition to the difficulty of establishing the diagnosis of sarcopenia.

Hypertensive etiology is responsible for the episode of HF decompensation in most patients, although those with normal muscle strength present an increase in other etiologies, such as ischemia. In previous studies, the etiology of HF is fundamentally ischemic in sarcopenic patients [14]. On the contrary, non-ischemic causes are more frequent in patients with normal muscle strength [14]. The included sample is mainly composed of patients with preserved LVEF, whose cause is not usually ischemic. The group of patients with reduced LVEF was a minority and responded to an ischemic etiology that usually affects younger patients, which is why it is associated in this sample with greater muscle strength.

Consistent with previous findings, mean LVEF was significantly lower in patients with normal muscle strength (48% vs. 52%). Again, it is exemplified that those with normal muscle strength have lower LVEF because they respond to an ischemic etiology but, at the same time, are younger and less comorbid, so their risk of sarcopenia is lower. These results seem similar to the literature, where patients with sarcopenia had better LVEF than non-sarcopenic patients [13,14].

In this study, the subgroup with low muscle strength had a worse functional class according to the NYHA scale. This is consistent with other articles in which sarcopenic patients also had worse functional class [14]. However, another study shows that patients with sarcopenia and HF with reduced LVEF do appear to have worse functional class, but not if they have preserved LVEF [13]. Nevertheless, given the subjective nature of the NYHA scale, these data should be interpreted with caution.

In the results obtained, 22 patients (5.84%) died during admission, together with 36 (9.55%) more in the following 12 months. Mortality figures during baseline admission are similar to Spanish ones in patients with acute HF, although a lower mortality rate at one year is observed [4]. In our study, higher mortality was observed in those with low muscle strength but without reaching statistical significance. However, other articles define sarcopenia and low muscle strength measured by dynamometry as risk factors for mortality [12,13]. In line with the above, a recently published meta-analysis on the prognostic role of handgrip in patients with heart failure [19] concludes that low muscle strength measured by dynamometry is associated with greater mortality during follow-up [19].

On the other hand, a non-significant greater tendency to readmission was also observed in those with low muscle strength. In the aforementioned meta-analysis, those with lower handgrip also had a higher risk of the composite endpoint of readmission and mortality during follow-up [19]. The small sample size of the study has probably limited the achievement of statistical significance in both aspects.

However, we have found that the Profund index is related to low muscle strength in a statistically significant and independent way. In our study, patients with low muscle strength have higher scores on the Profund index. This translates into a lower probability of survival after one year. Although there is no evidence in the literature on the relationship between this index and sarcopenia, it seems logical that they could be associated. Polypathological patients tend to be more malnourished and inflamed due to their underlying diseases. Also, they are less physically active due to their lower functionality and age. As we have seen, this is related to both HF and sarcopenia. We want to highlight this fact because the comprehensive management of these patients is essential to improve their quality of life and prognosis. In this study, the sample size is probably limited to reaching statistical significance in mortality and readmission rates, but the probability of survival for patients with lower muscle strength is lower.

The limitations of the study include the fact that it is an observational and retrospective study with the consequent accompanying biases. Likewise, the small sample size limits the generalizability of the results and reduces statistical power. Quality of life variables, which are important in the elderly population, were not included. Finally, only low muscle strength and not muscle mass were considered for the diagnosis of sarcopenia, given the low accessibility to the diagnostic tests necessary for quantification.

As strengths, we included elderly and polypathological patients, frequently excluded from clinical trials, providing evidence on aspects to be improved in our daily clinical practice. Sarcopenia and HF are two entities with common pathophysiological pathways, and their combined approach could improve the prognosis of these patients.

## 5. Conclusions

Elderly polypathological patients with acute heart failure and low muscle strength measured by dynamometry have a higher PROFUND index and, therefore, a lower probability of survival at one year. They also have worse functional class according to the NYHA scale. Sarcopenia is a prevalent disease related to heart failure, and we must manage both entities to improve the prognosis of these patients.

## Figures and Tables

**Table 1:** Descriptive analysis of the sample.

Variable	n = 377
Normal muscle strength	67 (17.77%)
Low muscle strength	310 (82.23%)
**Epidemiological variables**	
Sex (n, %)	
Men (n, %)	166 (44.03%)
Women (n, %)	211 (55.97%)
Age (mean, SD)	83 (±9)
**Anthropometric variables**	
Weight (kg) (mean, SD)	73 (±17)
Height (cm) (mean, SD)	160 (±9)
BMI (kg/m^2^) (median, RIC)	27 (24, 32)
**Comorbidities**	
Arterial hypertension (n, %)	343 (90.98%)
Diabetes mellitus (n, %)	177 (46.95%)
Dyslipemia (n, %)	245 (64.99%)
Atrial fibrillation (n, %)	286 (75.86%)
Chronic renal disease (n, %)	204 (54.11%)
Chronic respiratory disease (n, %)	156 (41.38%)
Cerebro-Vascular disease (n, %)	69 (18.65%)
Cognitive impairment (n, %)	37 (9.81%)
Neoplasia (n, %)	33 (8.75%)
Osteoarticular disease (n, %)	94 (24.93%)
**Clinical Variables**	
Heart failure etiology (n, %)	
Hypertensive (n, %)	154 (40.85%)
Ischemic (n, %)	75 (19.89%)
Dilated (n, %)	8 (2.12%)
Valvular (n, %)	86 (22.81%)
Amyloidosis (n, %)	14 (3.71%)
Others (n, %)	40 (10.61%)
Previous year hospital admission (n, %)	
0 (n, %)	56 (14.85%)
1 (n, %)	166 (44.03%)
2 (n, %)	87 (23.08%)
3 (n, %)	41 (10.88%)
4 (n, %)	16 (4.24%)
5 (n, %)	5 (1.33%)
6 (n, %)	5 (1.33%)
8 (n, %)	1 (0.27%)
Everest scale (mean, SD)	7 (±3)
NYHA functional class (n, %)	
I (n, %)	14 (3.71%)
II (n, %)	169 (44.83%)
III (n, %)	179 (47.48%)
IV (n, %)	15 (3.98%)
LVEF (%) (mean, SD)	52 (±13)
**Analytical Variables**	
Hemoglobin (g/dL) (median, IQR)	12 (10, 13)
Lymphocytes (×10^6^/L) (median, IQR)	800 (10, 1429)
Creatinine (mg/dL) (median, IQR)	1 (1, 2)
LDL (g/dL) (median, IQR)	73 (54, 93)
Albumin (g/dL) (median, IQR)	4 (3, 4)
NTproBNP (pg/mL) (median, IQR)	5500 (2956, 10,493)
CA-125 (U/mL) (median, IQR)	41 (20, 89)
**Functionality, nutrition and frailty variables**	
SPPB (median, IQR)	3 (0, 6)
Barthel (median, IQR)	75 (50, 95)
MNA-SF (median, IQR)	11 (9, 13)
PROFUND index (median, IQR)	6 (3, 10)
Rockwood	
Robust	5 (1.33%)
Well, without disease	8 (2.12%)
Well, disease controlled	75 (19.89%)
Vulnerable	78 (20.69%)
Light IADL dependence	77 (20.42%)
Help for IADL and BADL	119 (31.56%)
Dependent	13 (3.45%)
Unknown	2 (0.53%)
**Treatment**	
Furosemide maximal dose (mg/day) (median, IQR)	80 (60,120)
Corticoids (n, %)	99 (26.26%)
Protein supplements (n, %)	37 (9.81%)
Morphine (n, %)	50 (13.26%)
**Prognostic Variables**	
Death during admission (n, %)	22 (5.84%)
Death at 12 months (n, %)	36 (9.55%)
12 months readmission (n, %)	132 (35.01%)

**Legend**: SD: standard deviation; IQR: interquartile range; kg: kilogram; cm: centimeter, BMI: body mass index; m^2^: square meter; NYHA: New York Heart Association; LVEF: left ventricular ejection fraction; g: gram; dL: deciliter; L: liter; mg: milligram; LDL: low-density lipoproteins; NTproBNP: N-terminal pro-B-type natriuretic peptide; pg: picograms; CA-125: cancer antigen 125; U: unit; mL: milliliter; SPPB: short physical performance battery; MNA-SF: Mini-Nutritional Assessment Short Form; IADL: instrumental activities of daily living; BADL: basic activities of daily living.

**Table 2:** Comparative analysis according to muscle strength by dynamometry.

Variable	Low Muscle Strength (n = 310)	Normal Muscle Strength (n = 67)	*p*
**Epidemiological Variables**			
Sex (n, %)			*p* = 0.6
Men (n, %)	133 (42.90%)	33 (49.25%)	
Women (n, %)	177 (57.10%)	34 (50.75%)	
Age (mean, SD)	84 (±8)	78 (±10)	*p* < 0.001
**Anthropometric variables**			
Weight (kg) (mean, SD)	72 (±17)	78 (±16)	*p* = 0.016
Height (cm) (mean, SD)	160 (±9)	164 (±9)	*p* = 0.003
BMI (kg/m^2^) (median, IQR)	27 (24, 32)	29 (25, 32)	*p* = 0.4
**Comorbidities**			
Arterial hypertension (n, %)	285 (91.94%)	58 (86.57%)	*p* = 0.4
Diabetes mellitus (n, %)	147 (47.42%)	30 (44.78%)	*p* = 0.8
Dyslipemia (n, %)	200 (64.52%)	45 (67.16%)	*p* = 0.8
Atrial fibrillation (n, %)	237 (76.45%)	49 (73.13%)	*p* = 0.8
Chronic renal disease (n, %)	168 (54.19%)	36 (53.73%)	*p* > 0.9
Chronic respiratory disease (n, %)	128 (41.29%)	28 (41.79%)	*p* > 0.9
Cerebro-Vascular disease (n, %)	60 (19.35%)	9 (13.43%)	*p* = 0.5
Cognitive impairment (n, %)	37 (11.94%)	0 (0%)	*p* = 0.021
Neoplasia (n, %)	29 (9.35%)	4 (5.97%)	*p* = 0.6
Osteoarticular disease (n, %)	80 (25.80%)	14 (20.90%)	*p* = 0.6
**Clinical Variables**			
Heart failure etiology (n, %)			*p* = 0.15
Hypertensive (n, %)	132 (42.58%)	22 (32.84%)	
Ischemic (n, %)	55 (17.74%)	20 (29.85%)	
Dilated (n, %)	5 (1.61%)	3 (4.48%)	
Valvular (n, %)	74 (23.87%)	12 (17.91%)	
Amyloidosis (n, %)	13 (4.19%)	1 (1.49%)	
Others (n, %)	31 (10.00%)	9 (13.43%)	
Previous year hospital admissions (n, %)			*p* = 0.047
0 (n, %)	42 (13.55%)	14 (20.90%)	
1 (n, %)	133 (42.90%)	33 (49.25%)	
2 (n, %)	75 (24.19%)	12 (17.91%)	
3 (n, %)	34 (10.97%)	7 (10.45%)	
4 (n, %)	15 (4.84%)	1 (1.49%)	
5 (n, %)	5 (1.61%)	0 (0%)	
6 (n, %)	5 (1.61%)	0 (0%)	
8 (n, %)	1 (0.32%)	0 (0%)	
Everest Scale (mean, SD)	7 (±3)	7 (±3)	*p* = 0.4
NYHA functional class (n, %)			*p* = 0.016
I (n, %)	9 (2.90%)	5 (7.46%)	
II (n, %)	129 (41.61%)	40 (59.70%)	
III (n, %)	159 (51.29%)	20 (29.85%)	
IV (n, %)	13 (4.19%)	2 (2.99%)	
LVEF (%) (mean, SD)	52 (±12)	48 (±14)	*p* = 0.033
**Analytical Variables**			
Hemoglobin (g/dL) (median, IQR)	12 (10, 13)	12 (11, 13)	*p* = 0.4
Lymphocytes (×10^6^/L) (median, IQR)	800 (11, 1498)	700 (7, 1355)	*p* = 0.2
Creatinine (mg/dL) (median, IQR)	1 (1, 2)	1 (1, 2)	*p* = 0.6
LDL (g/dL) (median, IQR)	74 (55, 92)	70 (53, 93)	*p* = 0.7
Albumin (g/dL) (median, IQR)	4 (3, 4)	4 (3, 4)	*p* = 0.057
NTproBNP (pg/mL) (median, IQR)	5512 (2733, 10,153)	5417 (3278, 10,726)	*p* = 0.7
CA-125 (U/mL) (median, IQR)	39 (19, 89)	49 (27, 90)	*p* = 0.4
**Functionality, nutrition and frailty variables**			
SPPB (median, IQR)	2 (0, 5)	5 (2, 9)	*p* < 0.001
Barthel (median, IQR)	70 (45, 95)	95 (80, 100)	*p* < 0.001
MNA-SF (median, IQR)	10 (9, 12)	11 (10, 14)	*p* = 0.056
PROFUND index (median, IQR)	7 (3, 11)	3 (2, 5)	*p* < 0.001
Rockwood			*p* < 0.001
Robust	2 (0.65%)	3 (4.48%)	
Well, without disease	5 (1.61%)	3 (4.48%)	
Well, disease controlled	53 (17.10%)	22 (32.84%)	
Vulnerable	63 (20.32%)	15 (22.39%)	
Light IADL dependence	63 (20.32%)	14 (20.90%)	
Help for IADL and BADL	111 (35.80%)	8 (11.94%)	
Dependent	13 (4.19%)	0 (0%)	
Unknown	0 (0%)	2 (2.99%)	
**Treatment**			
Furosemide maximal dose (mg/day) (median, IQR)	80 (60,120)	120 (60, 160)	*p* = 0.047
Corticoids (n, %)	90 (29.03%)	9 (13.43%)	*p* = 0.033
Protein supplements (n, %)	33 (10.65%)	4 (5.97%)	*p* = 0.5
Morphine (n, %)	41 (13.23%)	9 (13.43%)	*p* > 0.9
**Prognostic variables**			
Death during admission (n, %)	19 (6.13%)	3 (4.48%)	*p* = 0.8
Death at 12 months (n, %)	32 (10.32%)	4 (5.97%)	*p* = 0.23
12 months readmission (n, %)	111 (35.81%)	21 (31.34%)	*p* = 0.27

**Legend:** SD: standard deviation; IQR: interquartile range; kg: kilogram; cm: centimeter, BMI: body mass index; m^2^: square meter NYHA: New York Heart Association; LVEF: left ventricular ejection fraction; g: gram; dL: deciliter; L: liter; mg: milligram; LDL: low-density lipoproteins; NTproBNP: N-terminal pro-B-type natriuretic peptide; pg: picograms; CA-125: cancer antigen 125; U: unit; mL: milliliter; SPPB: short physical performance battery; MNA-SF: Mini-Nutritional Assessment Short Form; IADL: instrumental activities of daily living; BADL: basic activities of daily living.

## Data Availability

The data presented in this study are available on request from the corresponding author. The data are not publicly available due to privacy restrictions.
